# CNA Landscape of HER2-Negative Breast Cancer in Anthracycline-Based Neoadjuvant Chemotherapy Regimens

**DOI:** 10.32607/actanaturae.20377

**Published:** 2023

**Authors:** M. K. Ibragimova, E. A. Kravtsova, M. M. Tsyganov, N. V. Litviakov

**Affiliations:** Cancer Research Institute, Tomsk National Research Medical Center of the Russian Academy of Sciences, Tomsk, 634009 Russian Federation; National Research Tomsk State University, Tomsk, 634050 Russian Federation; Siberian State Medical University, Tomsk, 634050 Russian Federation

**Keywords:** breast cancer, CNA landscape, anthracycline-based regimens, neoadjuvant chemotherapy, prognosis

## Abstract

Critical evaluation of how and when to include anthracyclines in preoperative chemotherapy is becoming more relevant in an era when the molecular genetic approach not only allows for the development of biologically targeted therapeutics, but also implies the ability to select the patients likely to benefit from certain cytotoxic agents. Changes in the copy number aberration (CNA) landscape of luminal B HER2- negative (HER2−) breast cancer (BC) during anthracycline-based neoadjuvant chemotherapy (NAC) regimens were studied in order to identify groups of potential CNA markers of objective response and CNA markers for predicting the development of hematogenous metastasis. Comparison of CNA frequencies depending on the response to NAC showed that objective response was observed in a larger number of deletions in the 11q22.3 and 11q23.1 loci (*p *= 0.004). Comparison of CNA frequencies in groups of patients after treatment showed that hematogenous metastasis was observed with a greater number of amplifications in the 9p22.2 locus (*p *= 0.003) and with a greater number of deletions in the 9p21.3 locus (*p *= 0.03). Potential predictive CNA markers of objective response and prognostic CNA markers of hematogenous metastasis in anthracycline- based NAC regimens have been identified.

## INTRODUCTION

Neoadjuvant chemotherapy (NAC) is considered the standard for the combination treatment of HER2- positive (HER2+) breast cancer (BC). At the same time, the treatment of localized ER+/HER2-negative (HER2−) BC, which is characterized by poorer chemosensitivity compared to other clinical BC subtypes, can be challenging [1]. Pathological complete response (pCR) rates in HER2-negative (HER2−) BC are low, while the presence of residual disease does not have the same prognostic value as in other clinical subtypes [2]. However, the rate of survival of patients who display complete or partial regression after NAC differs significantly from that of patients for whom the disease has stabilized or continues to progress [3, 4]. In this regard, in the case of HER2− BC, it is important that we search for predictive markers of complete and partial regression, in contrast to pCR markers in the triple-negative (TN) and HER2+ BC subtypes.

Several approaches to the treatment of HER2− and metastatic BC patients exist to date. However, no gold standard has been established for first-line treatment so far. Anthracycline- and taxane-based regimens are considered traditional systemic approaches to firstline chemotherapy and neoadjuvant therapy in this disease subtype [5].

The presence of toxic side effects for anthracycline- based NAC regimens (cardiotoxicity, leukemogenic effects, and secondary malignancies) [6, 7, 8], along with the central medical ethics principle of “not to do harm”, makes it extremely arduous to not only identify patients with the highest positive response to chemotherapy, but also find the systemic approach with the highest possible therapeutic index and minimal risk of significant long-term treatment-related toxicity.

In 2021, the National Comprehensive Cancer Network Guidelines removed the anthracycline-based therapy both from the list of preferred regimens for the treatment of early-stage HER2+ BC and from the “regimen of choice” category [9]. However, the results of meta-analyses available to date show that anthracycline use is justified in luminal B ER+/HER2− BC [10].

Thus, there are problems involved in selecting a NAC approach in HER2− BC. The question of when and in which HER2− BC patients a certain NAC regimen should be used requires further discussion.

Anthracycline-based regimens are an important component of BC treatment, especially in TN BC with a high risk of recurrence (regardless of axillary lymph node involvement) and HER2−/ER+ BC with axillary lymph node involvement. For this reason, it is necessary to search for biomarkers that predict the response to anthracyclines during NAC in BC, including both pCR and partial regression, which are associated with a favorable outcome.

In this work, we studied the changes in the copy number aberration (CNA) landscape of luminal B HER2− BC in the presence of anthracycline-based NAC regimens to identify groups of predictive CNA markers of objective (pCR + partial > 50% regression) response to treatment and potential CNA markers for predicting hematogenous metastasis.

## EXPERIMENTAL

**Material and methods**

The study included 35 patients aged 25–68 years (mean age, 49.3 ± 0.1 years (Mean ± SE)) with morphologically verified luminal B HER2−BC of the stages T1-4N0-3M0 (IIA–IIIB). Luminal B HER2− subtype was defined as ER +, PR + or −, Ki67 > 30%.

According to the Consensus Conference on Neoadjuvant Chemotherapy in Carcinoma of the Breast (April 26–28, 2003, Philadelphia, Pennsylvania), patients received 4–8 NAC courses using the following regimens: FAC (fluorouracil, doxorubicin, and cyclophosphamide) AC (doxorubicin, cyclophosphamide), and CAX (cyclophosphamide, doxorubicin, and xeloda). The effectiveness of preoperative chemotherapy was evaluated based on the WHO and International Union against Cancer criteria using ultrasound and/or mammography, which were performed before treatment, after two NAC courses, and before surgery. Complete regression (100% tumor reduction), partial regression ( > 50% decrease in tumor volume), stabilization ( < 50% decrease or > 25% increase in tumor volume), and progression ( > 25% increase in tumor volume) were recorded. All cases of complete regression were confirmed by morphological analysis. According to international recommendations, BC patients with disease course stabilization or progression are included in the group with no response to NAC, and patients with partial regression form the group with an objective response during preoperative chemotherapy. It is impossible to obtain tumor samples if the tumor went into complete regression after NAC.

*Table 1* presents the main clinical and morphological characteristics of the patients included in the study.

**Table 1:** Clinical and morphological characteristics of the breast cancer patients included in the study

Clinical and morphological characteristic		Number of patients (abs. number, %)
Age (years)	≤ 45	10 (28.6)
	> 45	25 (71.4)
Menstrual status	Invasive ductal carcinoma	20 (57.1)
	Invasive lobular carcinoma	3 (8.6)
	Invasive unspecified carcinoma	5 (14.3)
	Other type	7 (20.0)
Tumor size	T_1_	8 (22.8)
	T_2_	25 (71.4)
	T_3_	1 (2.9)
	T_4_	1 (2.9)
Lymphatic metastasis	N_0_	16 (45.7)
	N_1_	14 (40.0)
	N_2_	1 (2.9)
	N_3_	4 (11.4)
Histological type	Monofocal	23 (65.7)
	Multifocal	12 (34.3)
Response to NAC	Progression	1 (2.9)
	Stabilization	11 (31.4)
	Partial regression	23 (65.0)
Median follow-up, months (M ± SE)		80.5 ± 1.1 (min–max: 24–148)
	Metastasis rate	13 (37.1)
Median onset of metastasis, months (M ± SE)		45.7 ± 0.4 (min–max: 4–130)
	Recurrence rate	4 (11.4)
Median recurrence rate, months (M ± SE)		72.5 ± 1.5 (min–max: 52–107)

Tumor biopsy samples obtained before treatment under ultrasound guidance and surgical samples resected after NAC were used in the study. DNA was isolated from 35 paired BC tissue samples obtained from each woman before and after NAC.

Microarray analysis was carried out using the high-density Affymetrix CytoScan HD Array (USA). Sample preparation, hybridization, and scanning were performed using an Affymetrix GeneChip® Scanner 3000 7G (Affymetrix). The results were analyzed using Chromosome Analysis Suite 4.0 (Affymetrix).

A statistical analysis of the data was performed using the Statistica 8.0 package (StatSoft Inc., USA). The χ2 test was used to assess the differences between frequencies (http://vassarstats.net/index.html). The survival rate was analyzed using the Kaplan– Meier method and logrank test.

*Compliance with patients’ rights and bioethics principles.* The study was conducted in accordance with the 1964 Declaration of Helsinki (amended in 2013). The study protocol was approved by the Biomedical Ethics Committee of the Cancer Research Institute of the Tomsk National Research Medical Center of the Russian Academy of Sciences (protocol No. 1, 01/14/2013). All patients provided an informed consent to participate in the study.

## RESULTS

**Fig. 1:**
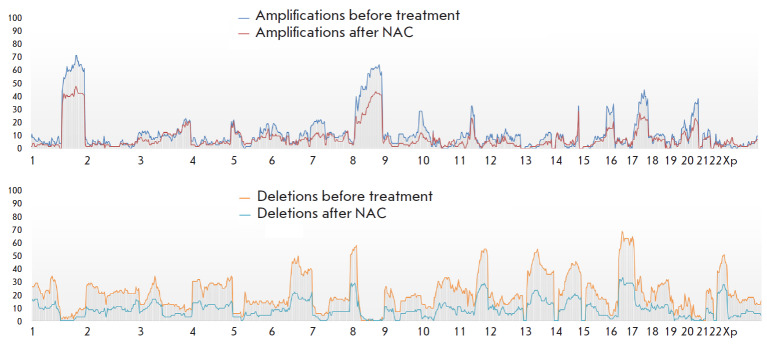
Frequency of amplifications and deletions in each chromosome in patients receiving anthracycline-based regimens before and after NAC as part of preoperative chemotherapy

At the first stage of the study, in order to evaluate the changes in the CNA landscape in the presence of anthracycline- based NAC, we described the tumor CNA landscape before and after treatment (*Fig. 1*) and assessed the changes in the CNA frequency in tumor.

The highest amplification number (68.6%) was found in the tumor loci 1q32.1–32.2, 1q42.12–42.13, and 1q42.2 in the patients before treatment. The highest deletion number (68.6%) was observed in the loci 17p13.3 and 17p13.1 (in the complete absence of amplifications). A total of 62.9% of amplifications were detected in the loci 8q21.3, 8q22.1–22.2, 8q23.3, 8q24.11–24.12m, and 8q24.21 on an extended region of the long arm of chromosome 8 in a complete absence of deleted regions.

The highest amplification number (48.6%) in tumor after treatment was noted in the loci 1q21.3, 1q32.1- 1q32.3, 1q41, 1q42.11–1q42.13, 1q42.2–42.3, 8q22.3, and 8q23.3; however, no deleted regions were found in the loci 1q21.3, 8q22.3, and 8q23.3. The highest rate of deletions (37.1%) was observed in the loci 16q21 and 16q22.1. A total of 34.3% of the deletions were detected in the tumor loci 11q23.3 and 17p13.3 after NAC, in a complete absence of amplified regions.

Analysis of the number of CNAs resulting from the use of anthracycline-based NAC regimens revealed a statistically significant decrease in the frequency of deletions in the loci 17p13.3 and 17p13.1: from 68.6 to 34.3%, which is 24/35 events before treatment and 12/35 events after NAC, respectively (*p *= 0.002).

*Figure 1* presents data on the amplification and deletion frequencies in each chromosome in BC patients before and after anthracycline-based NAC regimens.

Next, in order to search for potential CNA markers that could help predict an objective response to NAC during anthracycline-based regimens, we analyzed the distribution of CNA frequencies in tumor before treatment, depending on the response to preoperative chemotherapy.

Partial tumor regression was observed in 23 out of 35 patients (group 1) after treatment. Disease stabilization and progression were noted in 12 out of 35 patients (group 2) after therapy (*Table 1*).

Among group 1 patients, the highest amplification number (82.6%) was found in the loci 1q32.1–32.2, 1q42.12–42.13, and 1q42.2 in the absence of deletions. The highest deletion number (78.3%) was observed in the loci 11q23.1, 11q23.3, and 17p13.1 in the absence of amplifications. Among group 2 patients, the highest amplification number (58.3%) was detected in the loci 1q23.3, 8q21.11–21.13, 8q21.2, 8q21.3, 8q22.1–22.3, 8q23.1–23.3, 8q24.11–24.13, 8q24.21–24.23, and 8q24.3 in the absence of deletions. The highest number of deletions (59.0%) was recorded in 16q21 and 16q22.1 in the absence of amplifications.

Comparison of the CNA frequencies in these groups of patients demonstrated that an objective response to NAC was observed in the higher deletion number (18/23 events, 78.3%) in the loci 11q22.3 and 11q23.1 in group 1 compared to group 2 (3/12 events, 25.0%) (*p *= 0.004). *These loci could serve as predictive markers of objective response to anthracycline-based regimens in preoperative chemotherapy.*

In order to illustrate the complete picture of the tumor CNA landscape during treatment, we analyzed the distribution of CNA frequencies depending on the response to preoperative chemotherapy in patients after treatment.

Groups with partial regression (group 3, after NAC) and stabilization/progression (group 4, after NAC) were also formed.

In group 3 patients, the highest amplification number (47.8%) was observed in the loci 1q32.1–32.3, 1q41, 1q42.11–42.13, 1q42.2, 1q42.3, 8q22.3, and 8q23.3 in the absence of deletions; the highest number of deletions (34.8%) was found in the loci 8p23.2, 11q21, 11q22.1– 22.3, 11q23.1-23.3, 11q24.1, and 11q24.2.

Comparison of CNA frequencies in the group with partial tumor regression after anthracycline-based NAC regimens showed a statistically significant decrease in the amplification frequency, from 82.6% before treatment to 41.7% after treatment, in the loci 1q32.1 and 1q32.2 (*p *= 0.0001). Furthermore, the frequency of deletions in the loci 17p13.3 and 17p13.1 decreased after NAC (78.3 and 30.4% before and after treatment, respectively; *p *= 0.0002).

In group 4 patients, the highest amplification rate (75.0%) was found in the loci 1q21.3, 1q22, and 1q23.1– 23.3 in the absence of deletions; the highest number of deletions (58.3%) was observed in 16q21 and 16q22.1.

Comparison of CNA frequencies in the group with tumor stabilization and progression after anthracycline- based NAC regimens demonstrated an increase in the amplification rate in 6p12.2, from 0% to 25.0% before and after NAC, respectively (*p *= 0.001). The frequency of deletions in the loci 6p11.1 increased after NAC (0 and 25.0% before and after treatment, respectively;* p *= 0.001).

**Fig. 2:**
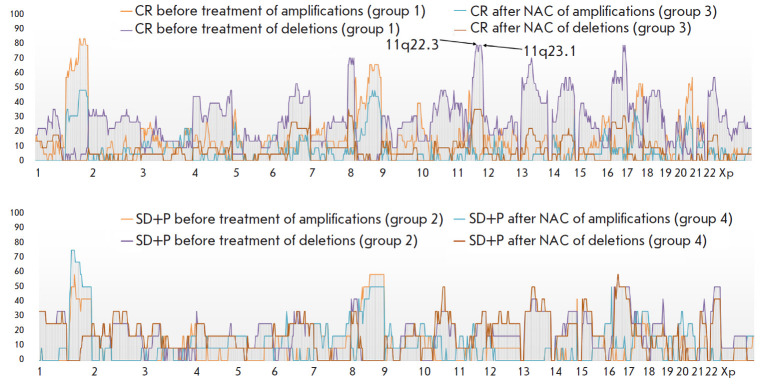
CNA frequency in breast cancer patients receiving anthracycline-based regimens before and after treatment as part of preoperative chemotherapy depending on the NAC effect. For group 1 and 3 patients with objective response to NAC (partial cancer regression), the CNA frequency before and after NAC is presented as amplification/deletion 1 and amplification/deletion 3, respectively. For group 2 and 4 patients with an absence of an objective response to NAC (cancer stabilization/progression, SD+P), the CNA frequency before and after NAC is presented as amplification/deletion 2 and amplification/deletion 4, respectively

*Figure 2* presents the summarized data on CNA frequencies in BC patients before and after therapy, depending on the NAC effect.

Next, in order to identify potential prognostic CNA markers of hematogenous metastasis during anthracycline-based NAC regimens, we analyzed the distribution of CNA frequencies in tumor before treatment, depending on the status of hematogenous metastasis.

Hematogenous metastasis was registered in 13 patients in the studied group. The following groups were formed: groups 5 and 6, which included patients without hematogenous metastasis before and after NAC, respectively, and groups 7 and 8, consisting of individuals with hematogenous metastasis before and after NAC, respectively. *Table 1* presents data on the rate of hematogenous metastasis and the median onset of metastasis.

In group 5, the highest number of amplifications (59.1%) was registered in the loci 8q21.3, 8q22.1–22.3, 8q23.1–23.3, 8q24.11–12, and 8q24.21 in the absence of deletions and in 1q32.1–32.2, 1q42.12–42.13, and 1q42.2 with a deletion rate of 9.1%. The highest deletions rate (77.3%) was found in 17p13.3–13.1 in the absence of amplifications.

In group 7, the highest amplification rate (84.6%) was detected in 1q23.2–23.3, 1q24.1–24.3, and 1q25.1– 25.3 in the absence of deletions. The highest deletion rate (76.9%) was observed in 11q23.3, 11q24.1, and 11q24.2 in the absence of amplifications.

Comparison of CNA frequencies in these groups showed that hematogenous metastasis took place in the highest amplification rate in the loci 18q11.2, 18q12.1, and 18q12.2. In particular, 23.0% of patients with diagnosed hematogenous metastasis had amplifications in these loci, while patients without hematogenous metastasis demonstrated no amplifications in these loci (*p *= 0.035).

In group 6 patients, the highest amplifications rate (54.6%) was found in the 1q21.3 locus, while no deletions were observed. The highest deletions rate (40.9%) was found in loci 16q21 and 16q22.1.

In group 8 patients, the highest amplifications rate (69.2%) was detected in 8q21.13, 8q21.2, 8q21.3, 8q22.1– 22.3, 8q23.3, 8q24.13, 8q24.21, and 8q24.22; the highest deletion rate (69.1%) was observed in 13q14.11–14.13 and 13q14.2, in the absence of amplifications.

Comparison of CNA frequencies in these groups after treatment showed that hematogenous metastasis is associated with a high number of amplifications in the 9p22.2 locus: 0/22 events (0%) and 5/13 events (38.5%) in the absence/presence of hematogenous metastasis, respectively (*p *= 0.002). Hematogenous metastasis was also developed in a greater number of deletions in 9p21.3 (0/22 events (0%) and 3/13 events (23.1%) in the absence/presence of hematogenous metastasis, respectively) (*p *= 0.053). *These loci could serve as prognostic markers of hematogenous metastasis in patients with luminal B HER2− BC receiving anthracyclinebased NAC regimens.*

**Fig. 3:**
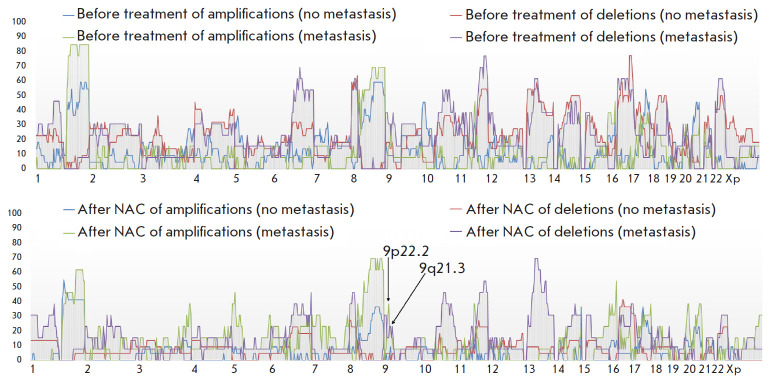
CNA frequency in breast cancer patients receiving anthracycline-based regimens before and after treatment as part of preoperative chemotherapy, depending on the presence of hematogenous metastasis

*Figure 3* shows CNA frequencies in BC patients receiving anthracycline-based regimens as part of preoperative chemotherapy before and after treatment, depending on the presence/absence of hematogenous metastasis.

To assess the metastasis-free survival (MFS) rate depending on the identified potential prognostic CNA markers of hematogenous metastasis after the use of anthracycline-based NAC regimens, we plotted survival curves using the Kaplan–Meier method.

**Fig. 4:**
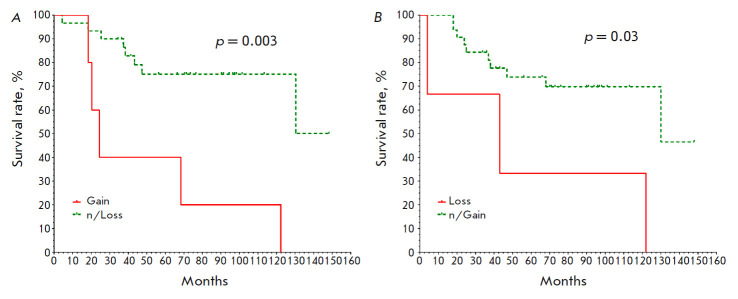
Non-metastatic survival rates of breast cancer patients depending on the presence of amplifications in the 9p22.2 locus (*A*) and deletions in the 9p21.3 locus (*B*) in tumor

*Figure 4* presents the curves of the BC patients included in the study, depending on the presence of 9p22.2 amplifications (*p *= 0.003) and 9p21.3 deletions (*p *= 0.03) in tumor.

## DISCUSSION

It is important to note that results of numerous studies on the search for prognostic and predictive markers in various adjuvant and neoadjuvant chemotherapy regimens against known molecular subtypes of BC have been published to date. The data of these studies are rather contradictory and aimed at a reevaluation of the use of available agents, including anthracyclines, while the attention is focused on the search for pCR markers.

In particular, centrosome duplication on chromosome 17 (*CEP17 *duplication) was studied as a marker of sensitivity to anthracyclines. An increased *CEP17* copy number is often found in BC [11, 12]. Analysis of BC samples for the presence of the *CEP17 *duplication at various rates (e.g. > 1.86 *CEP17*/cell, HER2/*CEP17* ≥ 2.0) was performed by several research groups and yielded contradictory results; either the presence or an absence of a linear relationship between the HER2/CEP17 ratio and pCR [11, 13, 14].

The dynamic changes in the HER2− BC genetic landscape were also studied. In particular, a series of prospective molecular profiling of HER2− BC tumors was conducted during chemotherapy. Tumor biopsies were obtained before and after 2 weeks of chemotherapy (doxorubicin/cyclophosphamide, ddAC). The tumor samples were obtained during surgery, after 8 weeks of combination therapy. To assess the single nucleotide variants (SNVs) and CNAs of 440 tumor- associated genes (ACTOnco®), NGS sequencing of the DNA of each patient (*n *= 34) was carried out at three time points. New mutations that developed due to therapy were found in 13% of cases (after one treatment cycle). A total of 72% of patients exhibited changes in variant allele frequencies (VAFs) of pathogenic SNVs: 51% of these changes developed early (after 2 weeks of therapy) and persisted for 8 weeks. The changes in SNV VAF were mostly associated with the PI3K/mTOR/AKT pathway. Tumors with a poor response to treatment (50% events, [7/14]) were less likely to develop SNV VAF compared to tumors with a good response (15% events, [4/24], *p *= 0.029). No significant difference in the CNA was noted between patients with a good and poor response after 2 weeks of therapy (22 [0–100] vs. 35 [0–106] events, respectively, *p *= 0.605). However, after 8 weeks, patients with a good response had a lower CNA load compared to patients with a poor response (12 [3–26] vs. 32 [15–73] events, respectively, *p *= 0.042) [15].

he results of an integrated multi-omics profiling of high-grade HER2− BC were presented. Identification of metastatic candidate driver events in stage III ER+HER2− tumors based on primary (*n *= 270) and metastatic diseases after treatment (*n *= 243) revealed amplification of 8q24.13 and 8q24.21 in 44.5% of metastatic cases [16].

We performed a search for potential predictive CNA markers of objective response to NAC and markers of hematogenous metastasis. In particular, we found deletions in the **11q22.3 **and **11q23.1 **loci as potential predictive markers of objective response to anthracycline-based NAC regimens as part of preoperative chemotherapy in patients with luminal B HER2− BC. Patients with deletions in these loci are statistically significantly more likely to develop an objective response to anthracycline-based NAC than those without deletions (*p *= 0.004).

In a study by Elin Barnekow et al., the **11q22.3 **locus is considered one of the eight most important BC susceptibility loci [17]. This locus was identified as a new risk locus (the most significant SNP is rs228595,* p *= 7 × 10-6) in *BRCA1 *mutation carriers [18]. The 11q22.3 locus contains several genes, including *ACAT1, NPAT*, and *ATM *(according to the data obtained using the genecards.org database).

It is important to note that recent studies indicate new and surprising functions for *ACAT1*, which encodes acetyl-CoA acetyltransferase. ACAT1 has lysine acetyltransferase activity, and it acetylates pyruvate dehydrogenase, which contributes to the Warburg effect and tumor cell proliferation [19]. According to recent data, ACAT1-mediated acetylation of METTL3 inhibits cell migration and invasion in TN BC [20]. It was also shown that inhibition of NPAT (nuclear protein, transcription coactivator) and p-NPAT prevents BC from entering the S phase of the cell cycle due to reduced DNA synthesis [21]. The common c.7271T>G mutation in *ATM *increases the risk of BC fourfold [22]. The role of *ATM *in BC has been studied in detail.* ATM *mutations have been found to correlate with certain clinical characteristics of BC such as lymph node involvement and HER2+ phenotype. *ATM *mutations are generally associated with a poor BC prognosis. In addition, since mutations in the *ATM*-encoding protein are involved in DNA repair mechanisms, *ATM* aberrations may also enhance the sensitivity of BC cells to platinum-based drugs and *PARP *inhibitors. Some data point to an association between *ATM *mutations and resistance of luminal positive BC cells to CDK4/6 inhibitors [23]. In our study, deletion of the* ATM *gene locus resulted in increased sensitivity of HER2− BC cells to anthracyclines.

We also showed that **9p22.2** and **9p21.3** could be considered prognostic makers of hematogenous metastasis in luminal B HER2− BC patients receiving anthracycline-based NAC regimens.

Both the amplifications in the **9p22.2** locus and deletions in the **9p21** locus (in contrast to its normal and amplified states) are considered unfavorable prognostic markers (*p*= 0.03).

Deletions of the short arm of chromosome 9 were shown to be associated with such aggressive BC signs as a highly malignant phenotype and a shortened survival time. The 9p deletions usually involve large fragments or even the entire chromosome arm [24]. The 9p21 deletions are associated with an unfavorable BC phenotype. In particular, 9p21 was found in 15.3% of 1,089 analyzed cases and associated with an unfavorable disease course, including a highly malignant phenotype (*p * < 0.0001), lymph node metastasis (*p *= 0.0063), and a high Ki67 index (*p * < 0.0001). The presence of the 9p21 deletion was associated with a poor disease outcome (*p *= 0.0720) [25].

According to published data, a homozygous deletion of the 9p21.3 locus is found in 15% of all human cancer diseases [26]. Recently, Han et al. analyzed largescale genomic data presented in the Cancer Genome Atlas (TCGA) and showed that the 9p21.3 deletion is a marker of a poor prognosis in several cancers, including BC. The study demonstrated a clear association between homozygous 9p21.3 deletion and shorter overall survival time [27].

## CONCLUSIONS

In the present study, we analyzed changes in the CNA landscape in breast cancer patients receiving anthracycline-based neoadjuvant chemotherapy regimens. We found potential predictive CNA markers of objective response (frequencies of deletions in loci** 11q22.3 **and **11q23.1**) and prognostic CNA markers of hematogenous metastasis (amplifications in the 9p22.2 locus and deletions in the 9p21.3 locus) in luminal B HER2− BC patients receiving anthracycline-based NAC regimens as part of preoperative chemotherapy. The obtained results are partially confirmed by the literature. However, validation of the obtained results is required in order to use the identified predictive and prognostic markers.

## Abbreviations

CNA: copy number aberration
BC: breast cancer
NAC: neoadjuvant chemotherapy
pCR: pathological complete response
SNV: single nucleotide variation
VAF: variant allele frequency
HER2: human epidermal growth factor receptor 2
MFS: metastasis-free survival
